# Rats’ (*Rattus norvegicus*) tool manipulation ability exceeds simple patterned behavior

**DOI:** 10.1371/journal.pone.0226569

**Published:** 2019-12-16

**Authors:** Akane Nagano

**Affiliations:** 1 Organization for Research Initiatives and Development, Doshisha University, Kyotanabe, Japan; 2 Faculty of Psychology, Doshisha University, Kyotanabe, Japan; University of Queensland, AUSTRALIA

## Abstract

Many studies have attempted to shed light on the ability of non-human animals to understand physical causality by investigating their tool-use behavior. This study aimed to develop a tool-manipulation task for rodents in which the subjects could not manipulate the tool in the direction of the reward by simple patterned behavior. Eight rats had to use a rake-shaped tool to obtain a food reward placed beyond their reach. During the training, the rats never moved the rakes laterally to obtain the reward. However, in the positional discrimination test, the rake was placed at the center of the experimental apparatus, and the reward was positioned on either the left or right side of the rake. Interestingly, this test indicated that some rats were able to manipulate the rake toward the reward without relying on a patterned behavior acquired during the training. These results suggested that rats have the primitive ability to understand causal relationships in the physical environment. The findings indicate that rats can potentially serve as an animal model to investigate the mechanisms of evolution and development of the understanding of physical causality in humans.

## Introduction

Some studies have suggested that non-human animals can understand causal relationships among multiple objects in the physical world [1, 2], suggesting that they can comprehend how the antecedent event A (the cause) produces the consequent event B (the effect), and not just understand the order of the two events; i.e., that event B always occurs after event A [2]. Many studies have investigated the tool-use behavior of non-human animals, including avian species and primates, to shed light on their understanding of physical causality [2]. However, further research using a wider variety of species in controlled experimental settings is needed to elucidate the mechanisms of evolution and development of the understanding of physical causality in humans.

In studies on rodents, including rats (*Rattus norvegicus*) [3, 4] and degus (*Octodon degus*) [5, 6], few have investigated the physical causal understanding in controlled experimental settings. In these previous studies, the subjects were commonly required to use a tool to obtain a reward beyond their reach. These studies suggested that rodents can understand the spatial and physical relationships between the tool and food [3–6]. However, to some extent, these tool-use tasks are problematic because the subjects could obtain the reward by using the tools through simple stimulus generalization (the transfer of a learned response from one stimulus to another similar stimulus [7]), simple trial-and-error learning (learning by accidental success which followed certain reactions of the subject [8]), or simple patterned behavior (behavior which was acquired through trainings by repeating the behavior for many times) rather than physical causal understanding.

Two types of tool-use tasks have been mainly used in previous studies on rodents: a tool-choice task and a tool-manipulation task [3–6]. In the tool-choice task, the subjects are required to choose a functional tool among multiple different shaped tools, including functional and non-functional tools, and pull the tool perpendicularly to obtain a food reward [3, 6]. In the test using this methodology, the subjects could choose the functional tools just by choosing the same or similar tools to the tool used to obtain the reward during training through simple stimulus generalization because a part of the tool was necessarily behind the reward in the correct option.

In the tool-manipulation task, the subjects are required to use a single rake-shaped tool to get the reward in situations where they cannot obtain it by pulling the tool perpendicularly toward them [4–6]. The previous studies on degus adopted a step-by-step protocol to gradually extend the distance between the rake and the reward [5, 6]. These two previous studies have reported that the degus could not obtain the reward by the tool in their first attempt in the initial phase, but that they gradually learned how to manipulate the tool [5, 6]. Especially in Okanoya et al. [6], the degus moved the tool back and forth and around the reward, pushing the tool or wiggling it horizontally. Thus, these studies indicated that the subjects could manipulate the rake to obtain the reward by simple trial-and-error learning [5, 6]. However, in a study on rats, the subjects were trained both by placing the rake on either the left or right side of the experimental apparatus, and by placing the reward at the side closer to the center of the apparatus than the rake in every trial; in contrast, during the test, the reward was placed on either the left or right side of the rake [4]. The results showed that six out of eight rats could manipulate the rake in the direction of the reward under the condition in which the rats could use only the position of the reward in relation to the tool as discriminative stimuli [4]. Thus, in this previous study [4], the subjects could manipulate the rake in the direction of the reward just based on simple patterned behavior, i.e., they moved the rake to the reward and pulled the rake perpendicularly toward the reward as they did during the training. Therefore, no research has revealed if rodents can understand physical causal relationships between manipulating tools toward the food and the food approaching them.

The objective of this study was to develop a tool-manipulation task for rodents in which the subjects could not manipulate the tool in the direction of the reward by simple stimulus generalization, simple trial-and-error, or simple patterned behavior. In this study, I provide basic performance data of rats subjected to the newly developed test. Specifically, I adopted the same procedures as Nagano and Aoyama [4]—including the number of trials, the types of food-reward, and the position of the rake to the reward—and analyzed rats’ performance data, which I could directly compare with the results of the previous study.

Unlike in Nagano and Aoyama [4], the rats never moved the rakes laterally toward the reward during the training sessions in this study; i.e., they never moved the rakes in the horizontal direction so that a part of the rake (the blade) and the reward were overlapped even just a little under conditions in which there was not a part of the rake behind the reward. Moreover, in the wild, rats are a non-tool-using species [9], and rats do not pull strings or tools to obtain food without training [3, 4, 10]. It can be assumed that rats do not have innate behavioral patterns to manipulate objects in the direction of the food. In the present study, I used experimentally naïve rats under a controlled setting. In addition, I divided the rats into two groups, which differed in the number of sessions and types of training trials because these parameters can affect their test performance.

## Materials and methods

### Subjects

Eight experimentally naïve three-month-old male Brown-Norway rats (subject numbers: BN41–BN48; Shimizu, Kyoto, Japan) were individually housed in wire cages. The rats weighed an average of 238.00 g (*SD* = 7.91) on the last day of free feeding. During training and testing, rats were maintained at about 85% of their free-feeding weight. However, all rats could gain approximately 10 g per month. The animal room was maintained under a 12:12-h light-dark cycle (light phase 8:00–20:00). All training and testing sessions were conducted during the light phase. All procedures and treatments were approved by the Doshisha University Animal Experiment Committee (protocol number: A16007) and were conducted in accordance with guidelines established by the Doshisha University Ethics Review Committee.

### Apparatus

The experiments were performed in an experimental box (outer dimensions: 21.0 cm wide × 21.0 cm long × 25.6 cm long) made of transparent acrylic boards, which was placed on a desk in the experimental room. A transparent sliding door, which the experimenter could open or close, was mounted at the front of the box. An experimental board (23.9 cm wide × 33.5 cm long), on which the tools and reward were presented, was set in front of the sliding door. Further details are described elsewhere [3].

**Fig 1:**
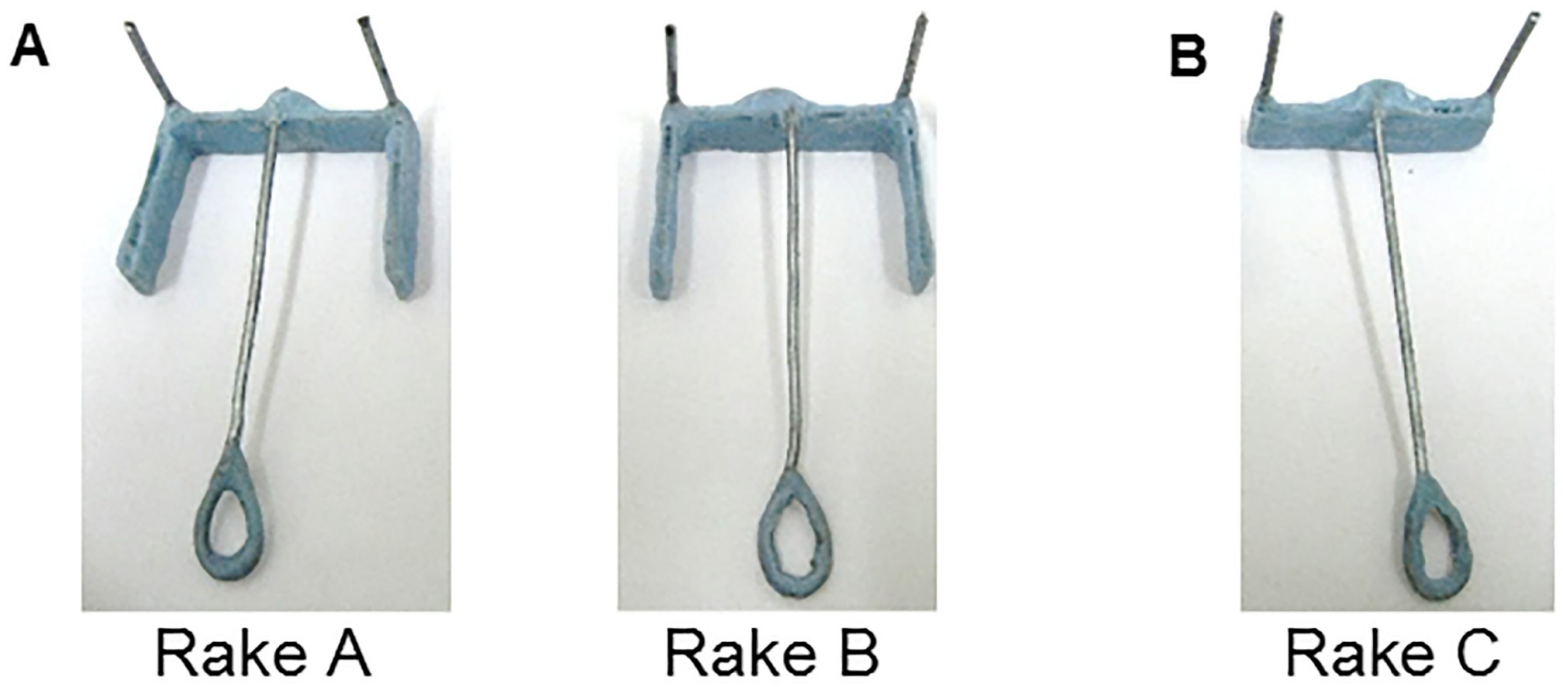
Different types of rake-shaped tools. (A) Rakes A and B used during training. These two rakes had guides on each end of the blade to prevent the reward from rolling out from the inside of the blade. (B) Rake C used in the positional discrimination test.

Three different rake-shaped tools (Rakes A, B, and C; Fig 1) were used. Each rake had a blade made from an acrylic board covered with resin for dental use (Ostron II Blue, GC Corporation, Tokyo, Japan) and a vertical wire pointing upward that was glued on each end of the blade. A handle (Rakes A, B, and C: 1.0 cm maximum wide × 7.0 cm long) made of wire and resin was glued to the blade center of each rake. Two rakes had the same shape (Rakes A and B: 4.0 cm maximum wide × 7.3 cm long × 3.2 cm maximum high, weight: 6.11 g; Fig 1A). A guide (Rakes A and B: 2.9 cm wide × 1.0 cm high × 0.2 cm thick) made of an acrylic board covered with resin was glued on each end of the blade. The third rake did not have the guides on the blade (Rake C: 3.8 cm maximum wide × 7.3 cm long × 3.2 cm maximum high, weight: 4.97 g; Fig 1B).

During the training and the testing, the subject’s behavior was recorded by a video camera (Panasonic, Japan, HDC-TM30) mounted above the experimental box. The experimenter sat at the front of the box, observed the subject’s behavior, and performed the following behavioral procedures.

### Procedures

#### Habituation

Before the training phase, rats were handled for 5 min per day for 5 days. The feeding restriction was introduced to control the subject’s weight on the third day of handling. From that day on, each rat was habituated to the food reward by receiving chocolate-flavored loops and brown-colored cereal (Ciscorn Sakusaku Ring, Nissin Cisco Co., Ltd., Osaka, Japan) in its cage for 5 days. The same cereals were used previously as rewards in tool-use task studies on rats [3, 4].

#### General training procedures

Three types of training sessions were performed: food-obtaining training, rake-pulling training, and rake-choice training. Daily experimental sessions comprised 40 trials each. An eighth to a sixth of a chocolate-flavored loop cereal was used as a food reward in each trial, as in Nagano and Aoyama [4].

#### Food-obtaining training

**Fig 2:**
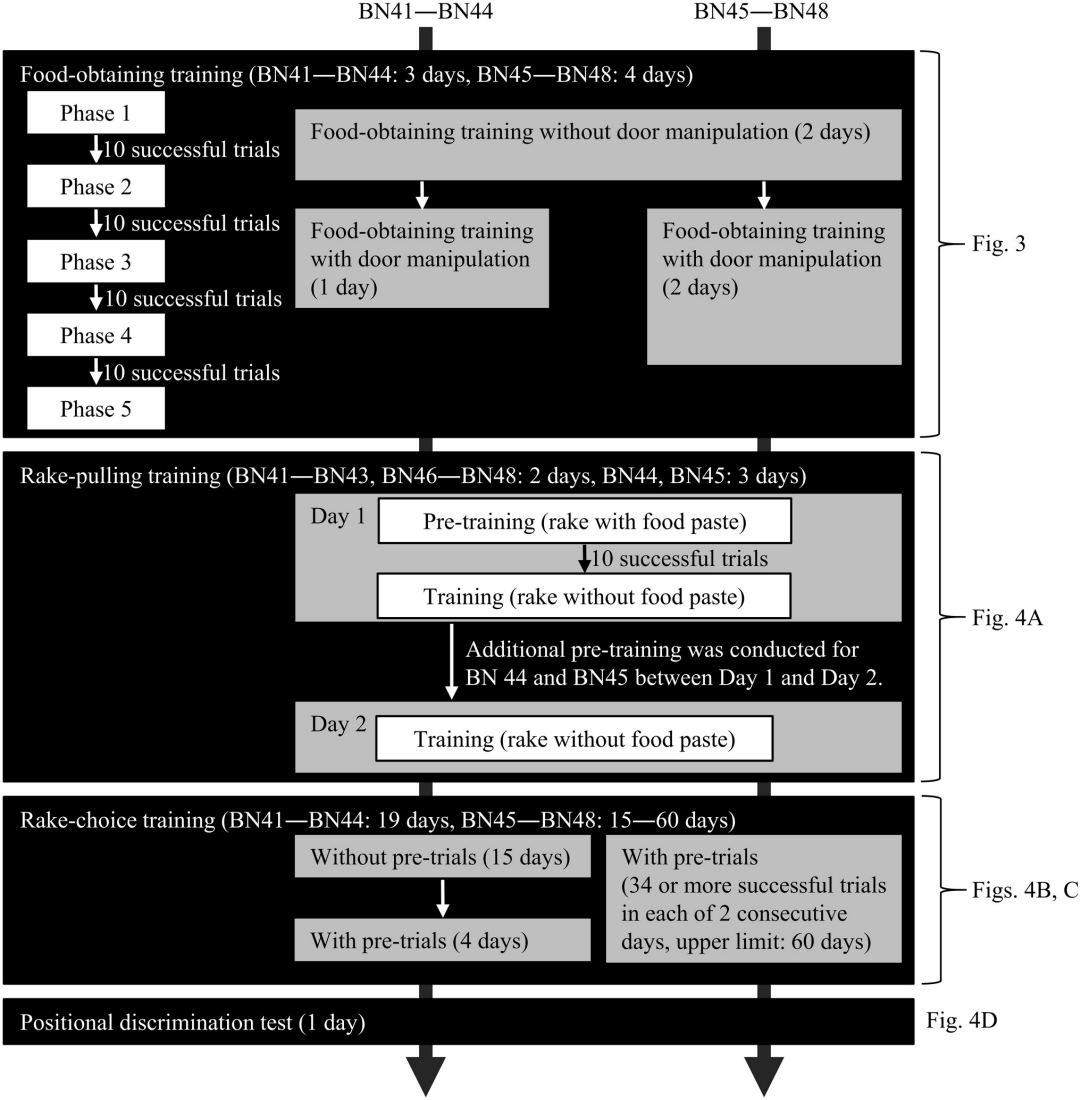
Flowchart of the food-obtaining training, the rake-pulling training, the rake-choice training, and the positional discrimination test. The operation of the sliding door of the experimental box was introduced irrespective of the phase that each rat reached. Each daily experimental session comprised 40 trials throughout this experiment.

**Fig 3:**
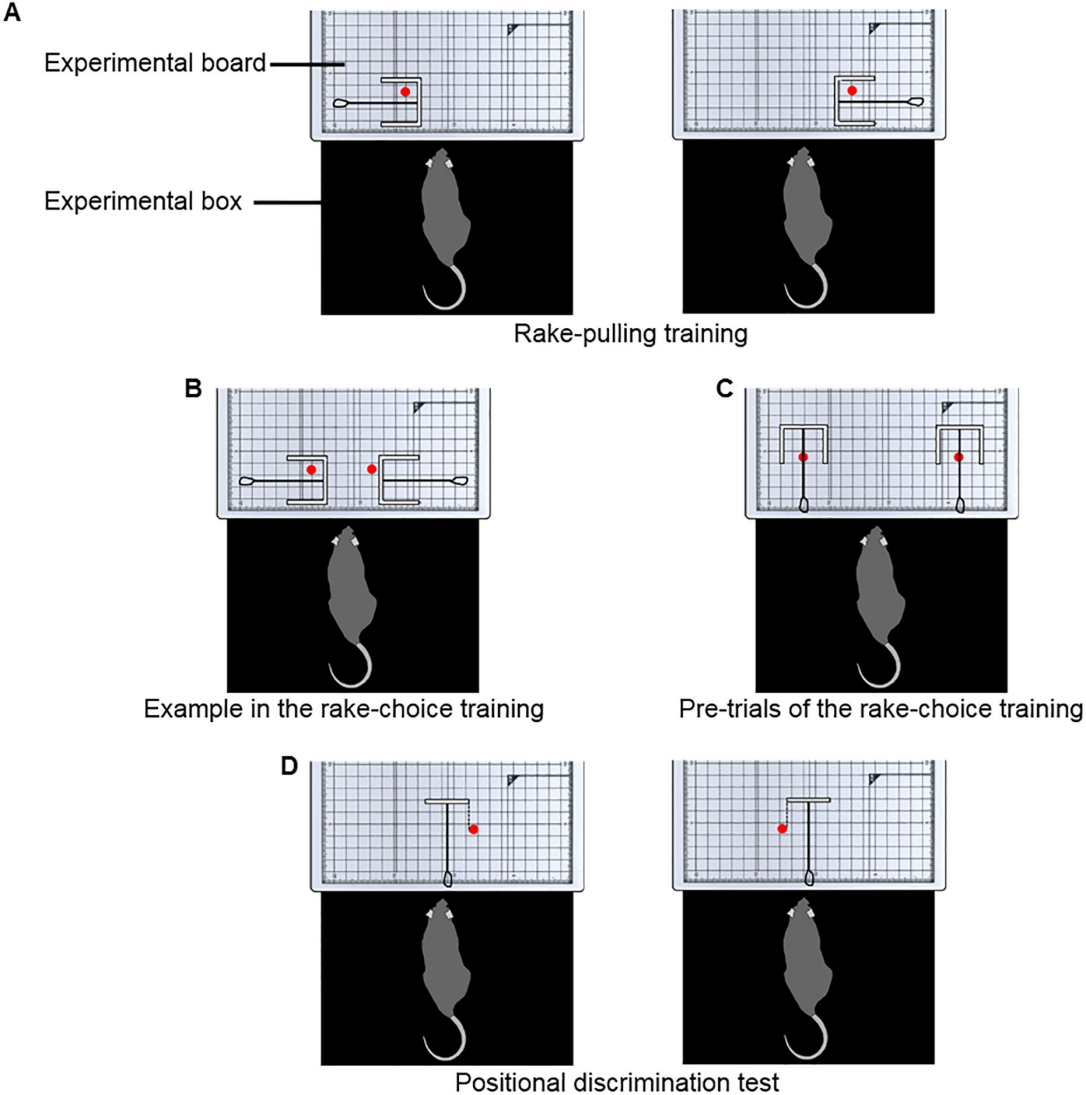
Arrangements of the reward in Phases 1, 2, and 5 of the food-obtaining training. Each red circle indicates the position of the reward.

In food-obtaining training, the rats first learned to obtain a piece of food with their paws or mouth (see S1 Appendix for details and S1 Movie). The purpose of this training was to develop their pulling behavior of Rakes A and B for the following rake-pulling training. In this training, the reward was placed within their reach inside the experimental box or on the experimental board that they could grab the reward with their paws or take it with their mouths. This training comprised five phases, and the distance between the reward and the rat was made increasingly longer by 1.0 cm (Figs 2 and 3).

#### Rake-pulling training

**Fig 4:**
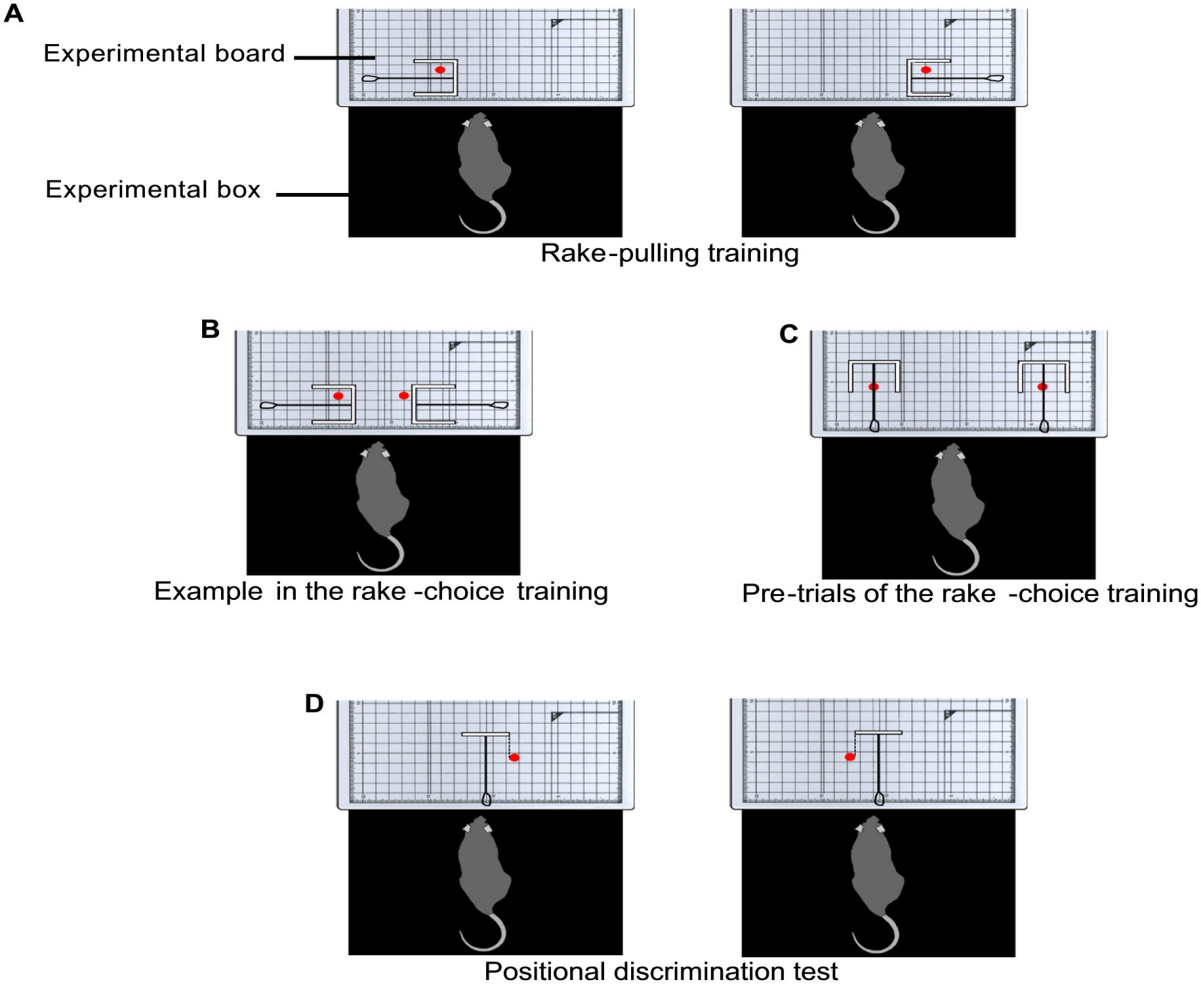
Arrangements of rakes and rewards during the rake-pulling training, the rake-choice training, and the positional discrimination test. Each red circle indicates the reward position. (A) The arrangements of Rake A or B and the reward in the rake-pulling training. (B) An example of the arrangement of Rakes A and B and rewards in the rake-choice training. (C) The arrangement of Rakes A and B and rewards in pre-trials during rake-choice training. (D) The arrangement of Rake C and the reward in the positional discrimination test.

In rake-pulling training, rats learned to pull the rakes with guides (Rakes A and B) to obtain the reward placed beyond their reach. The experimenter placed a rake with guides on the experimental board so that the handle of the rake was parallel to the sliding door and placed the reward inside the blade of the rake (Fig 4A). Thus, the reward was positioned on the side of the rake and prevented from rolling out from the inside of the blade by the guides. The experimenter used Rakes A or B randomly in each trial. This arrangement was adopted to stimulate the rats’ behavior of pulling the rake in various directions, not just perpendicularly. The door was opened 3 s after the experimenter placed the rake and the reward at the defined position on the board (trial start; Fig 4A). The tip of the handle of the rake was positioned at the same position as that of the reward in Phase 5 of the preceding food-obtaining training.

The rake-pulling training included a pre-training (S2 Movie). During pre-training, a paste mixture of the cereals from the rewards and some water was applied to the tip of the handle of the rake, and the experimenter presented the rake on the board. The application of the paste to the handle was adopted to promote the rats’ behavior of pulling the rake. If the rat pulled the rake and obtained the reward successfully in 10 trials (termination of the pre-training), the experimenter presented the rake without the paste in the following trials on the same day (S3 Movie). Both the rake and reward were placed on the side of the board, alternating between the left or right side. Each arrangement was adopted in one-half of the trials during each session in a pseudo-randomized order. All rats except for two (BN44, BN45) attained the criterion of the pre-training on Day 1. The experimenter conducted additional pre-training for these two rats for an additional day. After the pre-training, the experimenter conducted a training session with each rat using the rakes without the paste.

#### Rake-choice training

**Fig 5:**
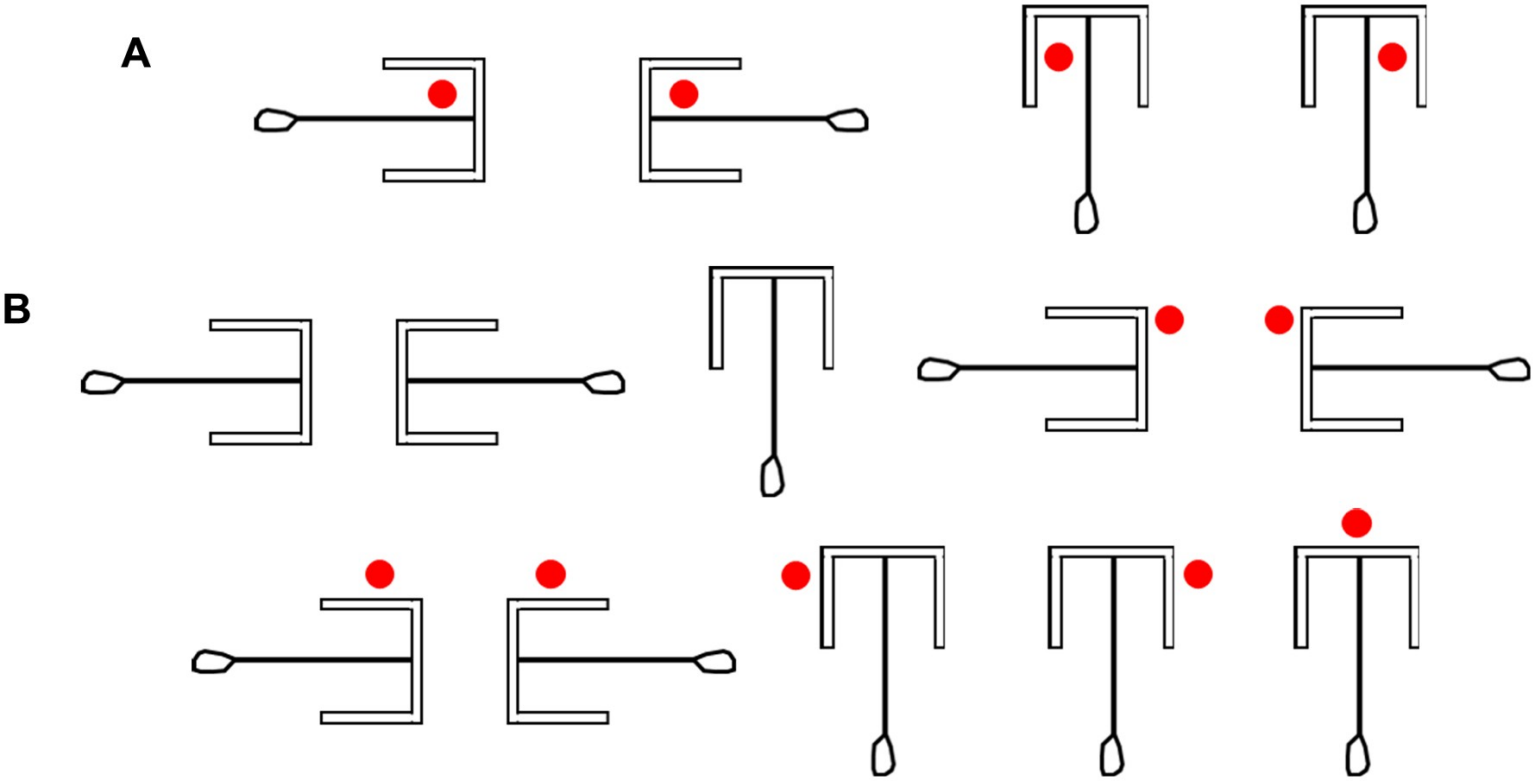
The spatial arrangements of the rakes and rewards in the rake-choice training. Each red circle indicates the position of the reward. (A) The spatial arrangements of the rakes and rewards of the appropriate options. (B) The spatial arrangements of the rakes and rewards of the inappropriate options. Regarding the first three options, the experimenter presented only the rake without presenting the reward.

In rake-choice training, rats had to choose between an appropriately arranged rake with guides and an inappropriately arranged rake with guides (see Fig 4B, S4 Movie, and S2 Appendix for details). If the rat pulled the appropriate rake perpendicularly, it could obtain the reward because the reward was placed inside the rake. However, if the rat pulled the inappropriate rake, it could not obtain the reward because the reward was placed outside the rake or no reward was placed. The experimenter presented Rakes A, B, and reward in 80 arrangements. There were 40 sets of appropriate and inappropriate options per rake that were obtained by combining four arrangements of the appropriate options and ten arrangements of the inappropriate options (Fig 5). Thus, the experimenter presented the rakes and the rewards in 80 arrangements by switching the position of the appropriate and the inappropriate options between the left and right sides. Each daily experimental session comprised 40 trials during this training. The 80 appropriate and the inappropriate arrangement options were presented in a pseudo-randomized order during two consecutive sessions. Rakes A and B were used in rake choice training, and the combination of rakes and appropriate or inappropriate options were randomized between trials.

The experimenter introduced a pre-trial immediately before Trial 1 in each session. In the pre-trial, the experimenter presented both Rakes A and B in an arrangement in which the rat could obtain the reward by pulling the rake perpendicularly; the arrangement was adopted only in the pre-trial and was not adopted in Trials 1 to 40. The pre-trial was introduced to prevent the development of a position preference in the rats that could result in choosing the rake from only one side. In the pre-trial, the door was opened 3 s after the experimenter placed the rakes and the rewards at the defined position on the board (trial start; Fig 4C). When the rat chose one of the rakes and obtained the reward, the experimenter retrieved the rake from this option; then, the experimenter retrieved the other rake and closed the door (trial end) when the rat chose the other rake and obtained the reward. The experimenter performed the Trial 1 procedure of the rake-choice training immediately after the pre-trial. For four rats (BN41–BN44), a pre-trial was introduced from Session 16, and a position preference was observed in one rat (BN44) during the rake-choice training. For the other four rats (BN45–BN48), a pre-trial was introduced from Session 1 to prevent any position preference prior to the trial.

For four rats (BN41–BN44), there were 19 rake-choice training sessions, irrespective of their performance. For the other four rats (BN45–BN48), the training continued until each rat had attained the criterion of 34 or more successful trials during each of two consecutive sessions. The criterion of the rake-choice training in the present study was almost the same level as that in a similar tool-choice training in the previous study on rats [3]. The upper limit in this training was 60 sessions.

#### Positional discrimination test

After the rake-choice training, the experimenter performed the positional discrimination test for one session using a procedure similar to that used in a previous study on rats [4]. In this test, the experimenter examined whether rats could manipulate the rake laterally in relation to the position of food without the experience of having performed this action during training. The rats were required to move the rake without guides laterally before pulling it to obtain the reward (S5 Movie).

At the beginning of the session, the rat was placed in the box with the sliding door closed. The door was opened (trial start) 3 s after the experimenter placed Rake C at the center of the experimental board and placed the reward on either the left or right side of the rake (Fig 1B). The reward was positioned so that the blade of the rake would not touch it even if the rat pulled the rake perpendicularly. Thus, the rats could use only the position of the reward in relation to the rake as a discriminative stimulus to determine the correct direction in which to manipulate the rake (Fig 4D). The distance between the blade of Rake C and the door was 7.7 cm, and the distance between the blade and the reward was 2.0 cm. Trials ended either when the rat had obtained the reward (successful trial) or after 1 min had passed (failure trial). The daily experimental session in this test comprised 40 trials.

#### Data scoring and analysis

Rats’ behavior was analyzed using video records from the positional discrimination test. The manipulation direction of the rake without guides was analyzed using the same method as Nagano and Aoyama [4], as described here briefly: In this test, when the rat manipulated the rake toward the reward, it was recorded as a correct-direction trial. However, when the rat manipulated the rake away from the reward, it was recorded as an incorrect-direction trial. These determinations were based on whether the intersection point of the blade and the handle of the rake was on the left or right side of the center-line of the experimental board when the rat pulled the rake 2.0 cm (i.e., to the horizontal line contacting the reward). The correct-direction rate is a behavioral index that would enable the detection of trials in which the subject understood the appropriate direction in which to move the tool to obtain the reward but was not successful because of insufficient motor ability [4]. Two trials were excluded from the analysis as the rat flipped the rake out of reach before pulling the rake 2.0 cm (two trials for BN44). For testing the reliability of the scoring method in the positional discrimination test, an independent coder analyzed 25% of the videos of the test. Cohen’s kappa for the manipulation direction of the rake was 0.80, indicating strong agreement between the coders [11].

In addition, the position of the nose of each rat was analyzed when it first touched the rake with the left or right paw in each trial based on the video records from the positional discrimination test. This analysis was conducted to investigate whether the position of the rat in relation to the rake and the reward influenced their correct-direction rate by using the same method as described previously [4]. Briefly, the first column was divided into 21 areas (Area 1–21) based on the squares on the experimental board (S1 and S2 Figs), and the position of the rat’s nose was recorded after trial initiation.

To analyze the relationship between the position of rats’ noses and the correct-direction rates in the positional discrimination test, the first column (Area 1–21) on the experimental board was divided into the left-side area to the handle of the rake (Area 1–10) and the right-side area to the handle (Area 12–21) with Area 11 at the center (S1 and S2 Figs). The number of trials in which each rat’s nose was in the left- or right-side area at the time of the first touch on the rake was calculated for each trial. Moreover, under the condition in which the reward was placed on the left side from the rat’s view (20 out of 40 trials), if the rat’s nose was in the left-side area, it was recorded as an ipsilateral trial. Under the same condition, if the rat’s nose was in the right-side area, it was recorded as a contralateral trial. Similarly, under the condition in which the reward was placed on the right side from the rat’s view (20 out of 40 trials), if its nose was in the right-side area, it was recorded as an ipsilateral trial. But if the rat’s nose was in the left-side area under the same condition, it was recorded as a contralateral trial. Both the ipsilateral and contralateral trials excluded the trials in which rats’ noses were located at the center (Area 11) on the board when they first touched the rake.

To examine the effect of their body parts used for pulling the rake on the performance in each trial in the positional discrimination test, their body parts used for pulling the rake were analyzed based on the video records. I recorded which body part (left paw, right paw, or mouth) the rats used when they pulled the rake on each trial. If the rats used multiple body parts during a trial, I compared the accumulated use time between each body part and recorded the body part used for the longest duration on each trial. In the following analyses, I adopted the number of trials in which the rats used each body part for the longest duration. Two trials were excluded from this analysis as the rat flipped the rake out of reach before pulling the rake 2.0 cm (two trials for BN44), as mentioned above. In addition, I analyzed whether rats used their left or right paws when they manipulated the rake in the left or right direction in the positional discrimination test. In half (20 trials) of the session of the positional discrimination test, the rats could obtain the reward by manipulating the rake to the left and in the other half (20 trials) by manipulating the rake to the right.

#### Statistical methods

In the rake-pulling training, the daily success rates were calculated by dividing the number of trials in which each rat obtained the reward within 1 min (the number of successful trials) by the total number of trials (40 trials per day).

In the rake-choice training, the choice rate of the appropriate rake was calculated by dividing the number of trials in which each rat chose the appropriate rake (number of appropriate rake choice trials) by the number of trials in which each rat chose either the appropriate or inappropriate rake (total number of choice trials). Moreover, I analyzed whether the rats chose the inappropriate rakes even in trials in which there was no reward for the inappropriate options (Fig 5B). The choice rate of the appropriate rakes in the trials in which the inappropriate options excluded the reward was calculated by dividing the number of trials in which each rat chose the appropriate rake in the trials by the number of trials in which each rat chose either the appropriate or inappropriate rake. To compare the average choice rates in Sessions 1 and 2, and the average choice rates in the second from the last and the last sessions, a paired *t*-test was performed using session as a within-subject factor.

In the positional discrimination test, the success rate was calculated for each rat. To compare the success rates in the last session of the rake-choice training and the session of the positional discrimination test, a paired *t*-test was performed using session as a within-subject factor. The correct-direction trials included trials in which the rat manipulated the rake in the correct direction but failed to obtain the reward. Moreover, the analysis of the positional discrimination test also determined whether the rats manipulated the rake without guides toward the reward (in the correct-direction) or not (in the incorrect-direction), and compared the number of trials in which the rake was moved in either direction (40 trials). The correct-direction rate of the rake manipulation was calculated by dividing the number of trials in which each rat manipulated the rake toward the reward (the number of correct-direction trials) by the number of trials in which each rat manipulated the rake in either direction. Using binomial tests, the number of correct- and incorrect-direction trials were compared for each rat. In the binomial tests, the null hypothesis was that the correct-direction rates are 50%. In addition, data analysis showed whether each rat manipulated the rake in the correct direction from the beginning of the session in the test. The daily sessions (40 trials) were divided into eight blocks to calculate the average correct-direction rate of rake manipulation, with each block consisting of five trials. Finally, to analyze whether there were correlations between the correct-direction rate in the positional discrimination test and the success rate on the last day of the rake-choice training, and between the correct-direction rate in the test and the number of rake-choice training sessions, performance data of the rats were plotted individually in each figure panel. Pearson’s product-moment correlation coefficients were calculated for these two sets of parameters.

To analyze the relationship between the position of rats’ noses and the correct-direction rates in the positional discrimination test, the ipsilateral trials were calculated for each rat. Using two-tailed binomial tests, the number of ipsilateral and contralateral trials was compared for each rat.

The rates of the ipsilateral paw-use trials in the test were obtained by dividing the number of ipsilateral-paw trials by the number of trials in which the rats manipulated the rake in either direction with their left or right paw. Moreover, I examined whether each rat’s correct-direction trials corresponded to the trials in which the reward was on the same side as their paws used for pulling the rake in the positional discrimination test. The concordance rates of these trials were calculated by dividing the number of trials in which the rats used the same side paw for pulling the rake as the position of the reward (the left or right side in the experimental board) by the number of the correct-direction trials. In the analyses, the trials in which the rats used their mouths were excluded. Moreover, the comparison between the number of ipsilateral paw- and contralateral paw-use trials, and the comparison between the number of trials in which the rats manipulated the rake in the correct direction with their ipsilateral and contralateral paws were conducted by using binomial tests.

The statistical analyses were performed using SPSS Statistics version 25.0. The criterion for statistical significance was set at *p* < 0.05.

## Results

### Rake-pulling training

On the day after the pre-training, all rats except one (BN45) learned to pull the rake with guides quickly to obtain the reward from Trial 1 or 2. The average success rate in the session (excluding the rate of BN45) was 96.79%. BN45 learned to pull the rake from Trial 16 during the session. The success rate of BN45 from its first success (Trial 16) to the final trial (Trial 40) was 96.00%.

### Rake-choice training

**Fig 6:**
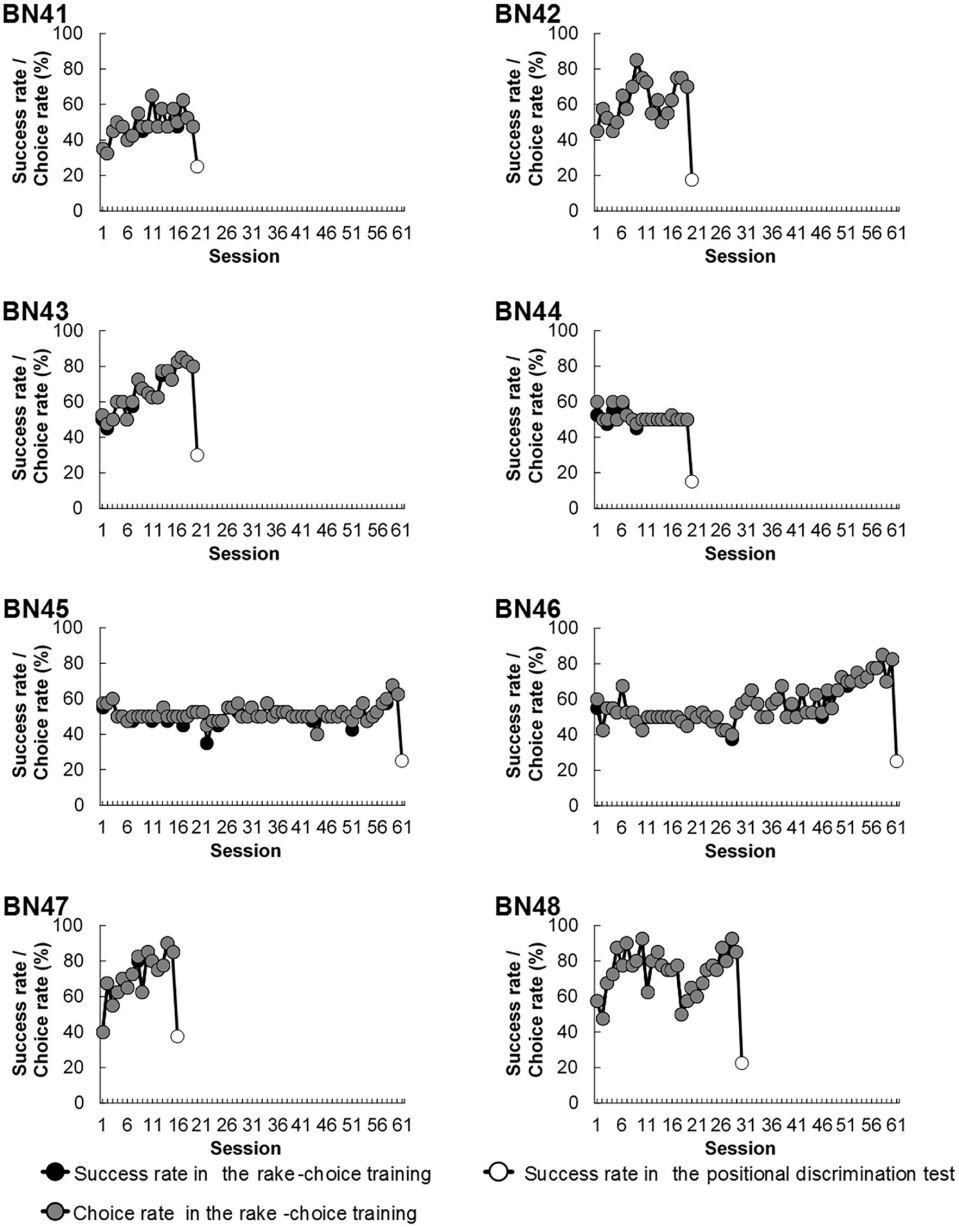
Individual (BN41–BN48) success and choice rates of the appropriate rake in the rake-choice training, and individual success rates in the positional discrimination test. Each black circle indicates the success rate during the training, and each white circle indicates the success rate in the test. Each gray circle indicates the choice rate of the appropriate rake in the positional discrimination test.

Out of four rats (BN41–BN44) without achievement criterion during the training, one (BN43) chose the appropriate rake and obtained the reward in more than 80% of trials within 19 sessions (Fig 6). However, the success rates of the other two rats (BN41, BN44) remained approximately 50%. The rate of the remaining rat (BN42) exceeded 80% temporarily until Session 9; then, it decreased rapidly before increasing again to 70% until the last session (Session 19; Fig 6). Moreover, among the four rats (BN45–BN48) trained with an achievement criterion, two rats attained the criterion in 15 (BN45) and 29 (BN48) sessions, whereas the other two rats did not meet the criterion within the 60 sessions (Fig 6).

The success rate and choice rate of the appropriate rake were identical in most sessions; however, in some sessions, the average success rate was slightly lower than the average choice rate because of a few trials in which the subject chose the appropriate rake but failed to obtain the reward (Fig 6).

Even in the latter sessions, some rats could not choose the appropriate rakes even in trials in which there was no reward for the inappropriate options (S3 Fig). There was no significant change in the choice rate of the appropriate rakes from the first two sessions to the last two sessions (*t* (7) = 1.67, *n*.*s*.; S4 Fig).

### Positional discrimination test

#### Performance

**Fig 7:**
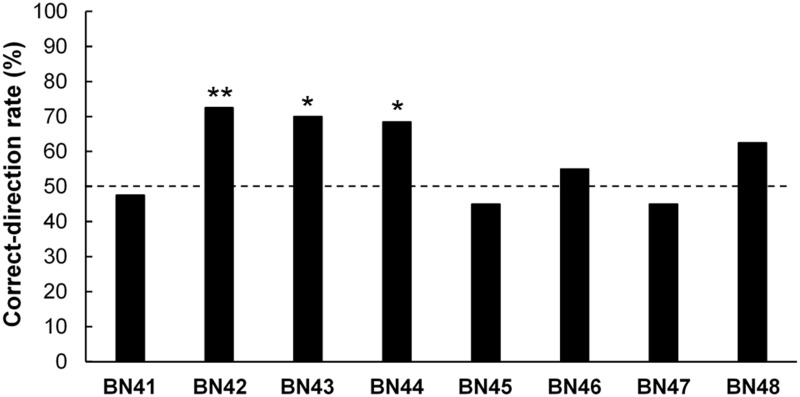
Individual (BN41–BN48) correct-direction rates in the positional discrimination test. The broken line indicates chance level (*p < 0.05, **p < 0.01).

The average success rate decreased significantly from the last session of the rake-choice training to the session of the test (*t* (7) = 9.82, *p* < 0.001; Fig 6). Three out of eight rats (BN42–BN44) manipulated the rake in the correct direction significantly more frequently than in the incorrect direction (Fig 7).

In addition, in the three rats (BN42–BN44) that showed a significantly higher rate of manipulating the rake in the correct direction, there was no tendency that the correct-direction rate increased gradually within a session (S5 Fig). The correlations were not detected between the correct-direction rate in the positional discrimination test and the success rate on the last day of the rake-choice training (*r* = 0.06, *n*.*s*.; S6A Fig), and between the correct-direction rate in the test and the number of rake-choice training sessions (*r* = -0.38, *n*.*s*.; S6B Fig).

#### The rats’ nose position at the time of the first touch on the rake

The position of the nose of each rat at the first time it touched the rake with the left or right paw in each trial was analyzed using the video records from the positional discrimination test. The purpose of this analysis was to investigate whether there was a possibility that the three rats (BN42–BN44) may have just moved to a position closer to the reward immediately before pulling the rake and, therefore, had the rake close to themselves for the pulling, which may have resulted in a correct-direction rate above the chance level (50%) during the test. For instance, perhaps the rats may have used a strategy that they moved to a position closer to the reward (left side) based on the position of the rake handle to try to obtain the reward with their paws and manipulate the rake in the left direction (correct direction). All rats had their noses on the ipsilateral and contralateral sides of the reward for the same number of trials when they first touched the rake (BN41–BN48: *n*.*s*.; Table 1).

**Table 1:** Individual rates of the ipsilateral trials in the positional discrimination test.

Subject number	Rate of ipsilateral trials (%)
BN41	50.00
BN42	50.00
BN43	67.86
BN44	50.00
BN45	52.78
BN46	50.00
BN47	50.00
BN48	38.46

The position of the nose of each rat was analyzed when it first touched the rake in each trial of the test. If the rat’s nose was on the same side of the rake handle as the reward, it was recorded as an ipsilateral trial. The cells filled with grey show the ipsilateral trial rates in the rats that manipulated the rake without guides in the correct direction significantly more frequently than in the incorrect direction in the positional discrimination test.

#### Body parts used for pulling the rake

I examined the effect of body parts used for pulling the rake on the performance in the positional discrimination test. All of the rats excluding one rat (BN44) used their right paw for the longest duration to pull the rake in most trials (S7A Fig). One (BN44) of the rats used its left paw in the majority of trials.

I conducted an additional analysis to determine whether rats used their ipsilateral paw to the reward when they manipulated the rake in the test. Two rats (BN43, BN45) used their ipsilateral paw more frequently than their contralateral paw, and the other two rats (BN41, BN47) used their contralateral paw more frequently (S7B Fig). In the other four rats (BN42, BN44, BN46, BN48), there were no significant differences between the numbers of trials in which they used their ipsilateral and contralateral paws for the longest duration (S7B Fig). Similarly, concerning the concordance rates between the correct-direction trials and the trials in which the reward was on the same side as their paws used for pulling the rake, the rates for two rats (BN43, BN45) was significantly above the chance level (50%) and the rates in the other two rats was significantly below the chance level. In the other four rats (BN42, BN44, BN46, BN48), the differences between the rates and the chance level were not significant (S7C Fig).

## Discussion

In the present study, I developed a tool-manipulation task for rodents in which the subjects could not manipulate the tool in the direction of the reward in the test by simple stimulus generalization, simple trial-and-error learning, or simple patterned behavior. The rats were tested in a controlled experimental setting. The results showed that some rats could manipulate the tool laterally according to the position of the food reward without the experience of having performed this action during the preceding training. In a previous study on tool-manipulation by rats, the subjects could manipulate the rake in the direction of the reward in the positional discrimination test only by manipulating the rake in a similar manner as in the preceding training [4]. In addition, previous studies that conducted the tool-manipulation task with degus found that the degus could manipulate the rake to obtain the reward after the step-by-step training as described above [5, 6]. Thus, the present study is the first to demonstrate that some rats can manipulate the tool in the direction of the food without being able to use a similar behavioral strategy based on manipulating the tool in the correct-direction in the test as in the training.

In the rake-choice training, the average choice rate of the appropriate rake did not generally change even in trials in which there was no reward for the inappropriate options. It would be possible that the rakes themselves turned to be the secondary reinforcers for the rats through their experiences in which they pulled the appropriate rakes and obtained the reward. Thus, the rats may have expected rewards from the rakes themselves and chosen between the rakes independent on the presence of the reward.

In the positional discrimination test, three out of eight rats manipulated the rake in the correct-direction significantly more frequently than in the incorrect-direction. These three rats (BN42–BN44) did not tend to increase the correct-direction rate within a session gradually. In addition, the finding that the ipsilateral and contralateral nose positions of the rats when they first touched the rake did not vary confirmed that these three rats did not use a simple strategy of moving closer to the reward immediately before they manipulated the rake to try to grab the reward with their paws and had pulled the rake toward themselves in the positional discrimination test. These findings suggest that rats can manipulate a tool according to the position of the reward, and they do it not just by simple patterned behavior. However, in five out of eight rats, the correct-direction rates in the positional discrimination test were not above chance.

What influenced the performance of the rats in the test? There was a difference in the procedure between the first four rats (BN41–BN44) and the remaining four rats (BN45–BN48). Interestingly, the rats that manipulated the rake in the correct-direction were from the first four rats. Therefore, it is possible that the difference in the procedure may have affected the correct-direction rate in the test. A pre-trial, in which the rats had to pull two rakes placed on the left and right side of the experimental board to obtain two rewards in total, was introduced from Session 16 for the former four rats (BN41–BN44) and from Session 1 for the latter four rats (BN45–BN48) in the rake-choice training. Thus, there was a change in the procedure between the sessions in the former four rats; however, there was no change in the latter four rats. The change of the procedure in the rake-choice training may have alerted them to the spatial arrangements of the tool and reward, and, as a result, this change may have stimulated their learning that obtaining the reward or not depended on the spatial arrangements of the tool and reward. Thus, the correct-direction rates in the positional discrimination test were above the chance level in the former four rats because they experienced the unexpected event that they could always obtain the reward by choosing either rake.

In the positional discrimination test, there were large individual differences in which body part was used to manipulate the rake, and the differences did not influence their performance. One rat (of the three rats that showed a significantly higher rate of manipulating the rake in the correct direction) used its ipsilateral paw more frequently than its contralateral paw to the manipulation direction of the rake and the position of the reward. In contrast, the other two rats used their dominant paw (BN42: right paw, BN44: left paw) more frequently than their non-dominant body part (BN42: left paw, BN44: right paw) independent of the manipulation direction of the rake and the position of the reward. This result was different from the previous study that has reported that rats tended to use their paw ipsilateral to the direction of rake manipulation more frequently than their contralateral paw [4]. It could be possible that the difference between the present study and Nagano and Aoyama [4] emerged from the difference between their experiences in these two studies.

Previous studies that investigated physical causal understanding in non-tool-using animals such as cotton-top tamarins (*Saguinus oedipus*) [12], rhesus monkeys (*Macaca mulatta*) [12], domestic cats (*Felis cattus*) [13], and Eurasian jays (*Garrulus glandarius*) [14], reported that by using an expectancy violation method the animals looked longer at an unexpected or a new event than at an expected or an original event. It is possible that rats look at or explore unexpected events in tool-use tasks because the tendency has been observed in a wide variety of non-tool-using species. However, further research is needed to identify the factors that can influence the performance in the test.

The success rates in the positional discrimination test were low in all the rats (15.0–37.5%). One possible factor is that the rake without guides was presented for the first time in the test. It is possible that the rats may have failed to obtain the reward even if they had manipulated the rake in the direction of the reward in the test because they may not have had the motoric capability to manipulate the novel rake laterally. In a previous study with rats, the rats were trained to manipulate a rake laterally during the training; the same rake was used in the positional discrimination test, and the success rates (approximately 35–75%) in the earlier test were higher than those in the test of the present study [4].

In addition, it is also possible that, even if the rats understood which direction (in the correct- or incorrect-direction) they had to manipulate the rake to obtain the reward, they may have adjusted to be more exploratory in manipulating it away from the food while repeatedly experiencing trials in which they had manipulated the rake toward the food and failed to obtain the food. Consequently, the correct-direction rate in the five out of eight rats (BN41, BN45–BN48) may not have been above chance. However, three out of eight rats (BN42–BN44) manipulated the rake in the correct direction at a high rate despite the low success rates. This result revealed that the rats did not manipulate the rake simply based on feedback concerning whether they could obtain the reward. If the rats simply manipulated the rake only based on the feedback, and they failed to obtain the reward in many trials even if they manipulated the rake in the direction of the reward, the number of the correct-direction trials should have been expected to decrease within the session.

In future studies, it would be necessary to improve the experimental procedure to shed light on physical causal understanding in rodents as follows: During the training, only the rake is presented in the experimental apparatus without presenting food rewards. The subjects are trained to manipulate the rake without guides laterally within fixed ranges, and an experimenter presents a reward to the subject by hand. After the training, the same positional discrimination test as the present study is carried out by using the same rake as that used during the training. During the training, the subjects never obtain rewards directly by using the rake, and the rake is never in contact with the food. Thus, when the subjects manipulate the rake laterally according to the reward’s position, it would be concluded that they can understand physical causality. It is because they cannot manipulate in the correct direction more frequently through simple stimulus generalization, simple trial-and-error learning, or simple patterned behavior based on their experience of the training.

The present study suggested that rodents may have a primitive ability to understand physical causality. Even by including similar studies with monkeys (*Macaca fascicularis*) [15], and 14–23-month-old human infants [16–19], the present study is the first to demonstrate that some subjects can manipulate a single rake-shaped tool based on the position of a reward in a test situation in which the reward is placed on the side of the tool for the first time. Especially in studies with human infants, the infants had many occasions to learn physical causal relationships between multiple objects in their daily lives (e.g., interactions with their parents, playing with toys, or eating a meal), and it can be assumed that they gained some experience in using tools with similar components as those presented in the experiments, even if the shapes and materials differed. In the present study, however, the subjects had fewer occasions to learn physical causal relationships between multiple objects, except for the tool-use task; therefore, the study subjects had no experimental experience before this experiment.

There were some limitations in the present study. In the rake-choice training, if the rats understood the rule that a part of the tool needed to be placed behind the food in the correct option, they could choose the appropriate rakes. In the positional discrimination test, the rats could manipulate the rake in the correct direction if they understood that the spatial arrangements of the blade of the rake and the food would be the same as those in the correct options of the rake-choice training by manipulating the rake toward the reward. However, it cannot be concluded from the present study whether the three rats which showed the significant correct direction rates in the positional discrimination test understood such complex physical causalities, but it may be possible that these three rats understood some primitive physical causalities. This possibility should be addressed in future studies.

Secondly, the analyses in the present study were based on a relatively small sample size. Thus, it would be possible that the correlations between the correct-direction rate in the positional discrimination test and the success rate on the last day of the rake-choice training, and between the correct-direction rate in the test and the number of rake-choice training sessions could not be detected because of lack of statistical power. In addition, the sample size would be too small to generalize to the results in the present study to rats in general. Therefore, these results have to be interpreted with caution, and future studies with a bigger sample size are needed for the generalization of the results.

Recently, many studies have attempted to shed light on the developmental mechanism of physical causal understanding by investigating tool-use behavior in animals [3–6, 20–38]. In the present study, I used naïve rats in an experimental setting. Rats are one of the most commonly used species in experiments in many research fields, currently third behind mice and fish [39], and controlling the conditions of tasks is relatively simple. Thus, rats are one of the most useful rodent models for investigating physical causal understanding. Studies with a variety of animal species subjected to the same positional discrimination test under multiple training conditions as in the present study would answer the following question—what factors of animals’ experiences can affect the acquisition of an ability to understand physical causal relationships, and how do animals develop this ability? In short, studies using a variety of naïve and non-tool-using species in experimental settings that include multiple training conditions would be useful to understand not only the mechanisms of evolution but also the underlying mechanisms for developing the ability of physical causal understanding. Thus, I propose using the rat as a rodent model to investigate the mechanisms of evolution and development of the understanding of physical causality in animals and potentially humans.

## Supporting information

S1 Appendix Details of the procedures in the food-obtaining training. (PDF)

S2 Appendix Details of the procedures in the rake-choice training. (PDF)

S1 Fig Individual (BN41–BN44) results on the number of trials in which the rat’s nose was located at each area in each trial in the positional discrimination test. The left panel indicates the results when the reward was placed on the left side of the rake; the right panel, the results when the reward was placed on the right side of the rake. Each broken line indicates the position of the handle of the rake.
(PDF)

S2 Fig Individual (BN45–BN48) results on the number of trials in which the rat’s nose was located at each area in each trial in the positional discrimination test. The left panel indicates the results when the reward was placed on the left side of the rake; the right panel, the results when the reward was placed on the right side of the rake. Each broken line indicates the position of the handle of the rake.
(PDF)

S3 Fig Individual (BN41–BN48) choice rates of the appropriate rakes in the trials in which the inappropriate options excluded the reward in the rake-choice training. The broken line indicates chance level.
(PDF)

S4 Fig Average choice rates of the appropriate rakes in the trials in which the inappropriate options excluded the reward in Session 1–2, and the second from the last and the last sessions of the rake-choice training. The broken line indicates chance level. Error bars indicate standard errors.
(PDF)

S5 Fig Individual (BN41–BN48) change in the correct-direction rates in the positional discrimination test. The broken line indicates chance level.
(PDF)

S6 Fig The relationship between the correct-direction rate in the positional discrimination test and the success rate or the number of sessions in the rake-choice training. (A) Individual results on the relationship between the correct-direction rate and the success rate. The vertical axis shows the correct-direction rate in the positional discrimination test, and the horizontal axis shows the success rate on the last day of the rake-choice training. (B) Individual results on the relationship between the correct-direction rate and the number of training sessions. The vertical axis shows the correct-direction rate in the positional discrimination test, and the horizontal axis shows the number of sessions in the rake-choice training.
(PDF)

S7 Fig Individual (BN41–BN48) results on body parts used for pulling the rake in the positional discrimination test. (A) Individual results for the number of trials in which each body part was used for the longest duration in the test. (B) The individual rates of ipsilateral paw-use trials in the test. Trials in which each rat used its paw for the longest duration were excluded from the analysis. The broken line indicates chance level (**p < 0.01, ***p < 0.001). (C) The individual concordance rates between the correct-direction trials and the trials in which the reward was on the same side as their paw used for pulling the rake for the longest duration in the test. Trials in which each rat used its paw for the longest duration were excluded from the analysis. The broken line indicates chance level (*p < 0.05, ***p < 0.001).
(PDF)

S1 Dataset Individual success and choice rates in the rake-choice training. (XLSX)

S2 Dataset Individual correct-direction rates in the positional discrimination test. (XLSX)

S3 Dataset Individual rates of the ipsilateral trials in the positional discrimination test. (XLSX)

S1 Movie Food-obtaining training. (MP4)

S2 Movie Pre-training in rake-pulling training. (MP4)

S3 Movie Rake-pulling training. (MP4)

S4 Movie Rake-choice training. (MP4)

S5 Movie Positional discrimination test. (MP4)
