# Supplementary material for: Rats’ (Rattus norvegicus) tool manipulation ability exceeds simple patterned behavior
Source: PLoS One. 2019 Dec 16;14(12):e0226569. doi: 10.1371/journal.pone.0226569 (PMC6913977; doi:10.1371/journal.pone.0226569)
Supplement: S1 Appendix — (PDF) [file pone.0226569.s001.pdf]

S1 Appendix. Details of the procedures in the food-obtaining training

At the beginning of the session, the rat was placed in the box. Irrespective of the phase that each rat had reached, the sliding door of the experimental box was kept open on Day 1 and Day 2 during this training so that the space between the end part of the sliding door and the surface of the experimental board was 1.7 cm. A trial started when the experimenter placed the reward at the defined position on the board (Fig. 3).

In Phase 1, the reward was placed inside the box directly on the board (Fig. 3). During this phase, the trial ended either when the rat started eating the reward or when 1 min had passed. If the rat started eating the reward within 1 min, it was recorded as a successful trial; however, if the rat did not start eating the reward within 1 min, it was recorded as a failed trial and the experimenter retrieved the reward.

During Phases 2 to 5, the reward was placed on the experimental board. In Phase 2, the experimenter placed the reward so that the distance between reward and sliding door was 0 cm (Fig. 3). The distance was increased by 1.0 cm every time the rat reached the criterion of the previous phase (Fig. 3). During these four phases, the trial ended when the rat had either obtained the reward or when 1 min had passed. If the reward from the board entered the inside of the box, it was recorded as a successful trial. In contrast, if the rat could not obtain the reward within 1 min, it was recorded as a failed trial and the experimenter retrieved the reward.

From Day 3 of the food-obtaining training, the experimenter introduced the operation of the sliding door irrespective of the phase that each rat had reached. At the beginning of the session, the rat was placed in the box with the door closed. The door was opened 3 s after the experimenter placed the reward at the defined position on the experimental board (trial start; Fig. 3). The space between the end part of the door and the surface of the board was 1.7 cm. In successful trials, the experimenter closed the door immediately after the rat obtained the reward. During failed trials, the experimenter closed the door after 1 min and retrieved the reward.

In each session, the reward was placed on the side of the board, alternating between the left or right side. Each arrangement was adopted for one-half of the trials in each session in a pseudo-randomized order. Each daily experimental session consisted of 40 trials in this training. During Phases 1 to 4, 10 successful trials resulted in the advancement to the next phase. In Phase 5, the rats were required to complete 10 successful trials to meet the success criterion. During Phases 1 to 5, 10 non-consecutive successful trials could meet each criterion. The food-obtaining training continued for each rat until it met the criterion for Phase 5. Three or four days were required to train the rats in obtaining the reward successfully (BN41–BN44: three days; BN45–BN48: four days).
