# Supplementary material for: Rats’ (Rattus norvegicus) tool manipulation ability exceeds simple patterned behavior
Source: PLoS One. 2019 Dec 16;14(12):e0226569. doi: 10.1371/journal.pone.0226569 (PMC6913977; doi:10.1371/journal.pone.0226569)
Supplement: S2 Appendix — (PDF) [file pone.0226569.s002.pdf]

**S2 Appendix.** Details of the procedures in the rake-choice training

At the beginning of the session in the rake-choice training, the rat was placed in the experimental box with the sliding door closed. The sliding door was opened 3 s after the experimenter placed rakes and reward(s) at the defined positions on the experimental board (trial start; Fig. 4B). The space between the end part of the door and the surface of the board was 1.7 cm. When the rat touched the rake with its left paw, right paw, mouth, or nose and the rake moved even just a little, it was regarded as choosing the rake. If the rat chose the appropriate rake, the experimenter retrieved the rake and reward of the inappropriate option immediately and then retrieved the appropriate rake immediately after the rat had obtained the reward before closing the door (successful trial). If the rat chose the inappropriate rake, the experimenter retrieved the rake and reward of the appropriate option immediately and closed the door after 30 s (failure trial). The experimenter also retrieved the rake and reward of the inappropriate option immediately before the door was closed. In addition, if the rat chose neither the appropriate nor the inappropriate option after 1 min had passed, the experimenter retrieved the two rakes and rewards of both options and closed the door (no-choice trial). As for three incorrect options without reward (Fig. 5B), the experimenter retrieved only the rake.

Four rats (BN41–BN44) were subjected to 19 rake-choice training sessions, irrespective of their performance. The other four rats (BN45–BN48) continued the training continued until each rat had attained the criterion of 34 or more successful trials during each of two consecutive sessions. The upper limit number of sessions in this training was 60 sessions. Consequently, the experimenter performed this training for 15 sessions with BN45, for 60 sessions with BN46 and BN47, and for 29 sessions with BN48.
