# Supplementary figures and images for: Rats’ (Rattus norvegicus) tool manipulation ability exceeds simple patterned behavior
Source: PLoS One. 2019 Dec 16;14(12):e0226569. doi: 10.1371/journal.pone.0226569 (PMC6913977; doi:10.1371/journal.pone.0226569)

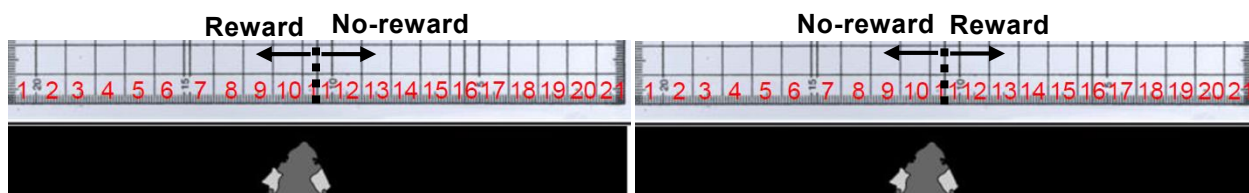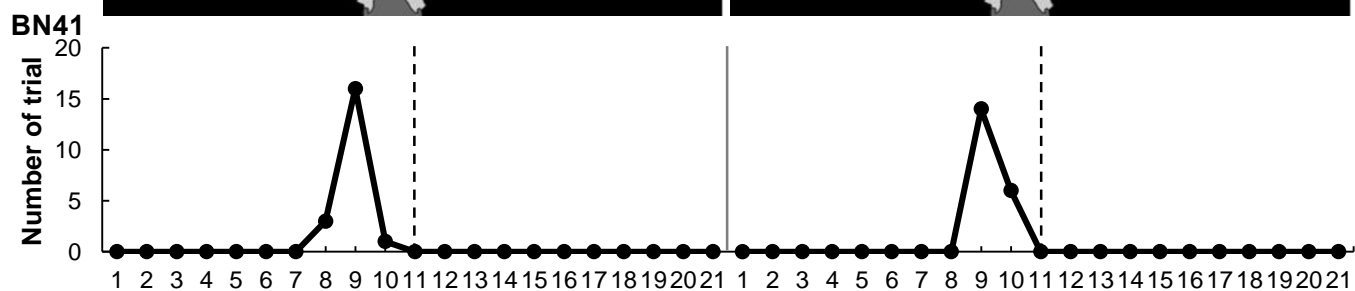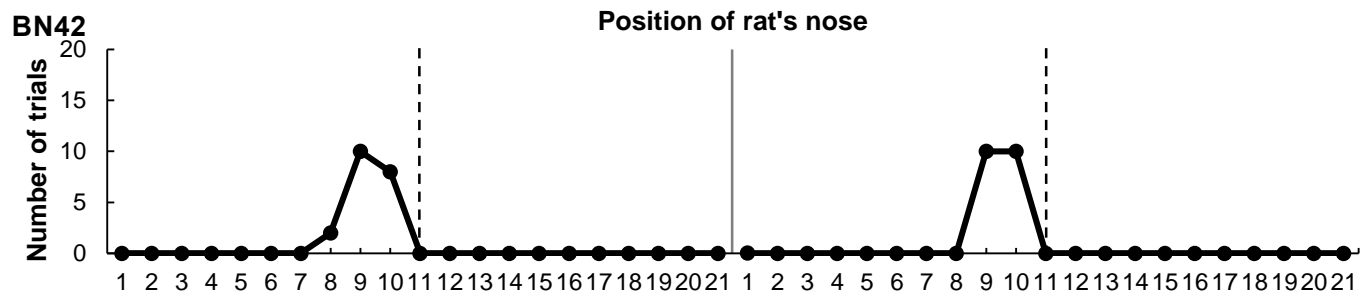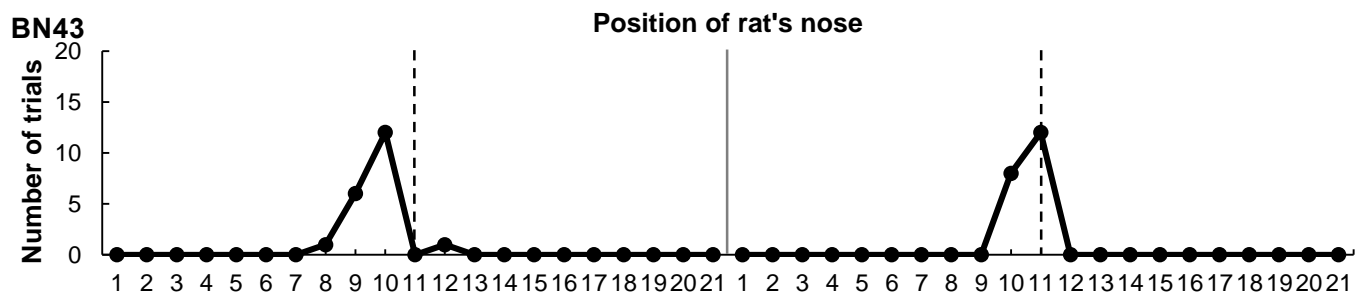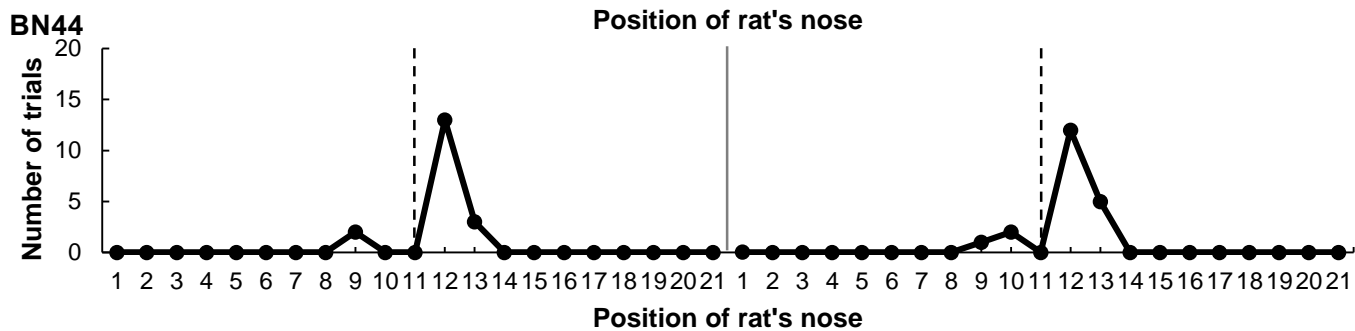

Supplement: S1 Fig — The left panel indicates the results when the reward was placed on the left side of the rake; the right panel, the results when the reward was placed on the right side of the rake. Each broken line indicates the position of the handle of the rake. (PDF) [file pone.0226569.s003.pdf]

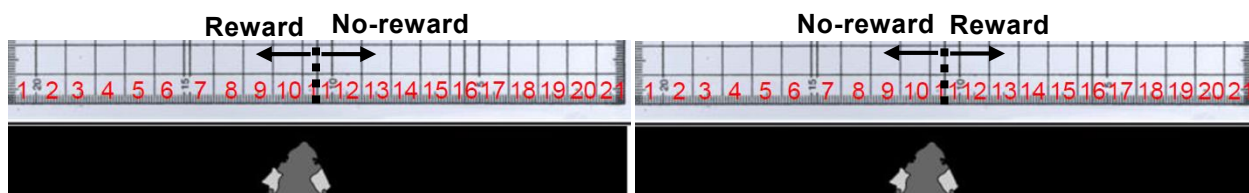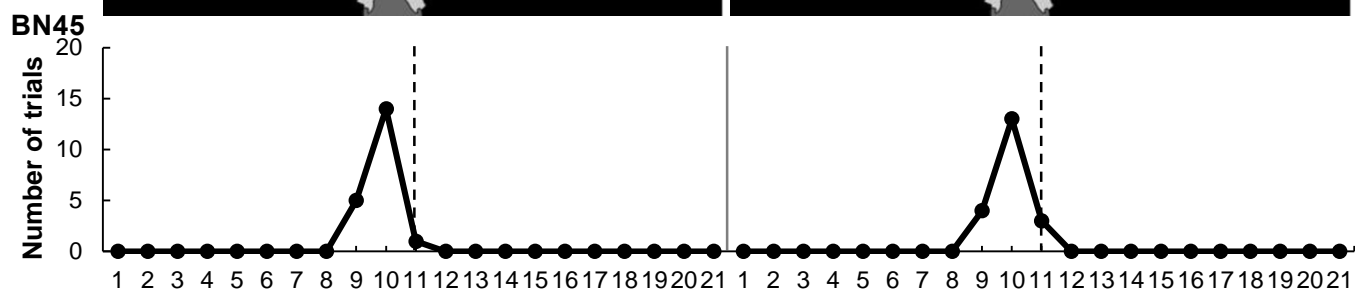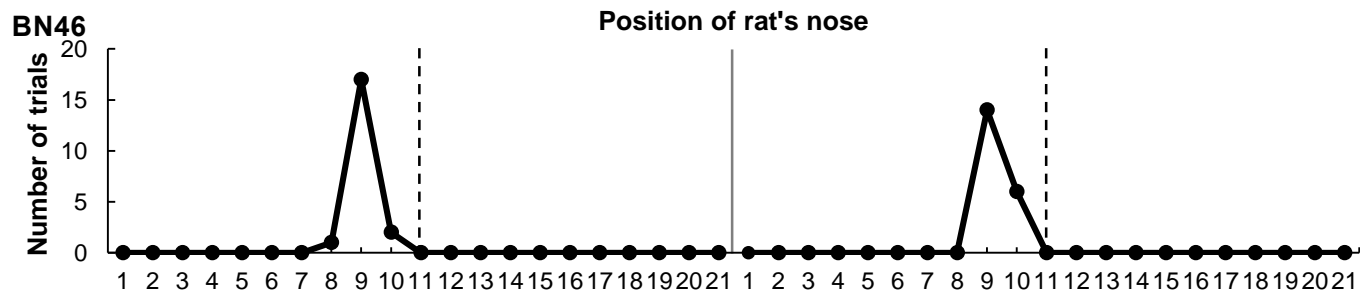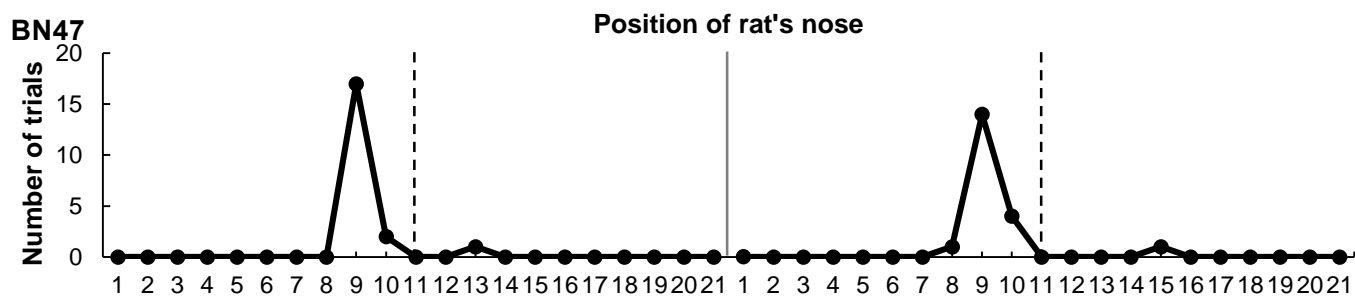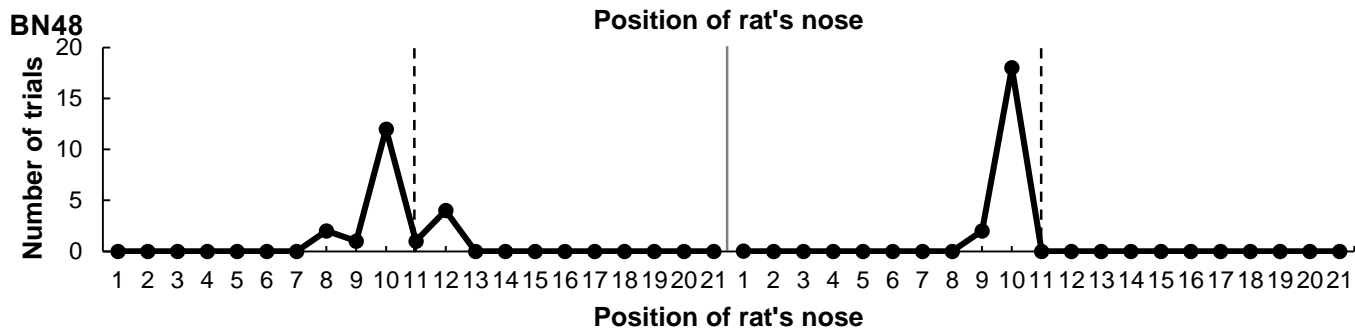

Supplement: S2 Fig — The left panel indicates the results when the reward was placed on the left side of the rake; the right panel, the results when the reward was placed on the right side of the rake. Each broken line indicates the position of the handle of the rake. (PDF) [file pone.0226569.s004.pdf]

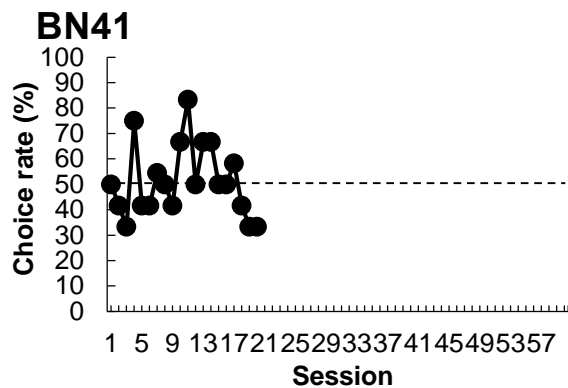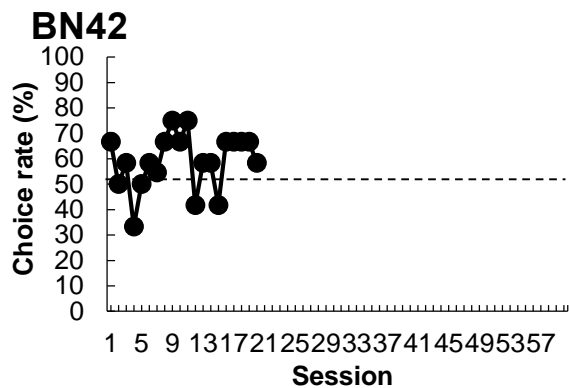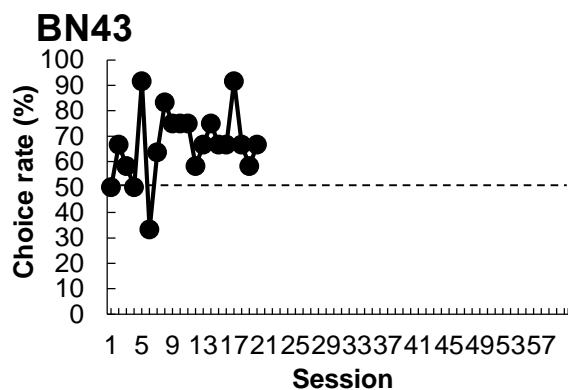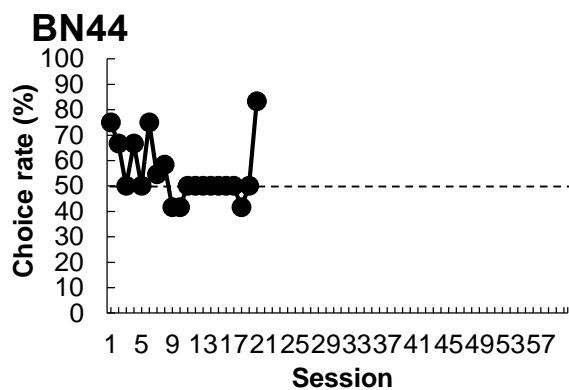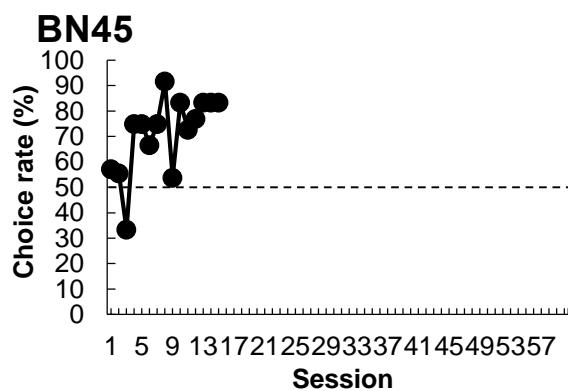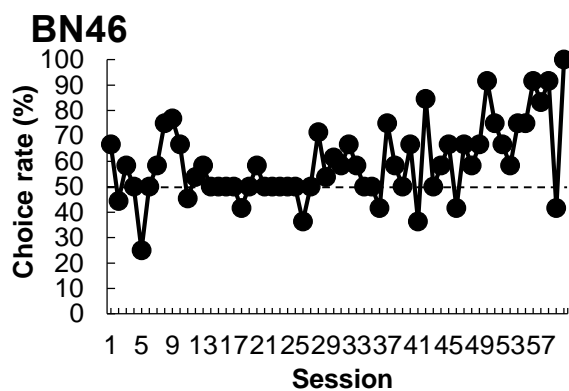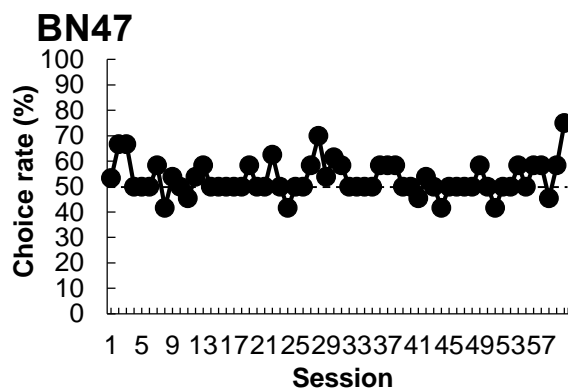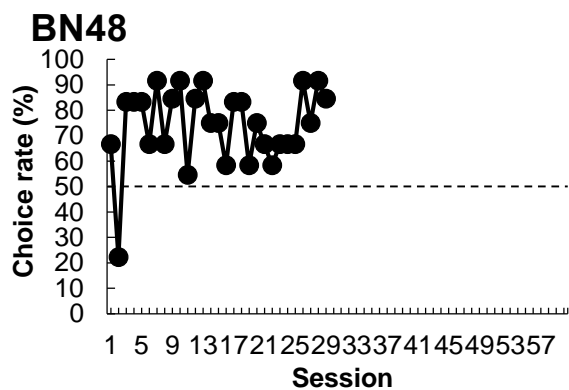

Supplement: S3 Fig — The broken line indicates chance level. (PDF) [file pone.0226569.s005.pdf]

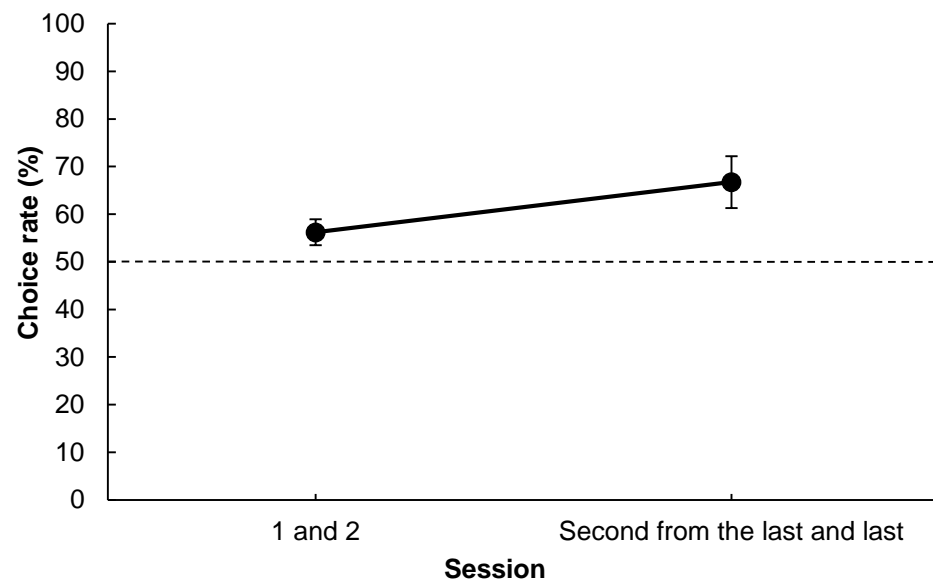

Supplement: S4 Fig — The broken line indicates chance level. Error bars indicate standard errors. (PDF) [file pone.0226569.s006.pdf]

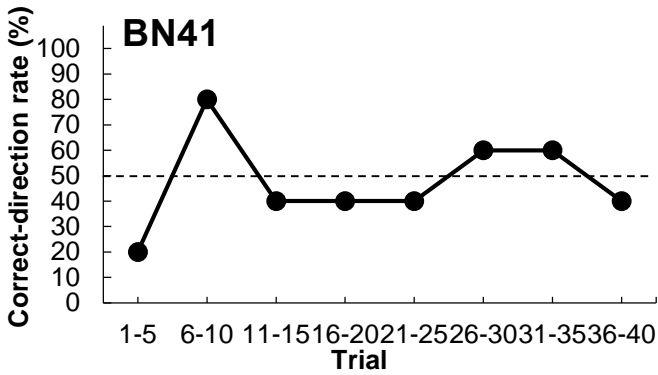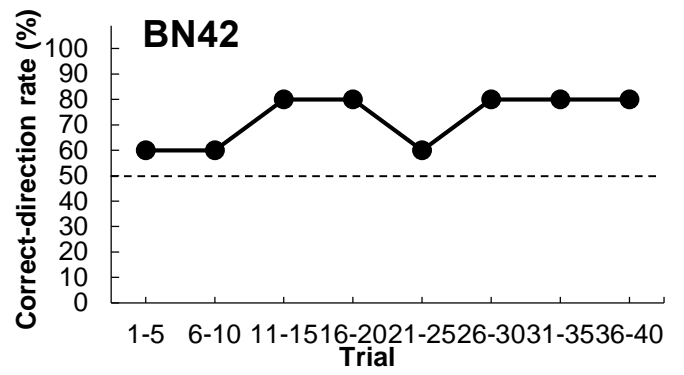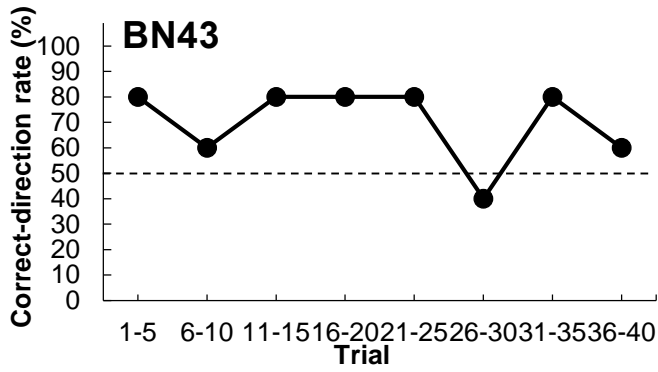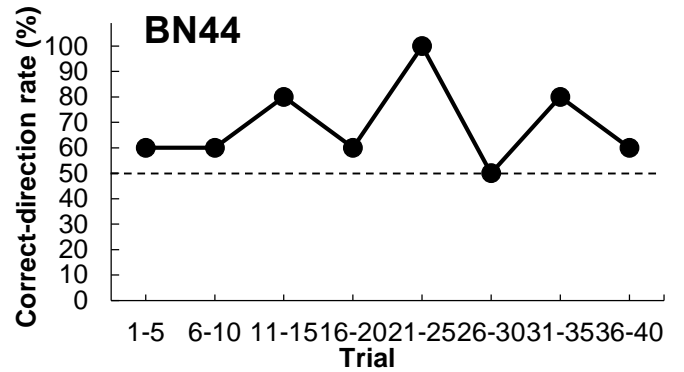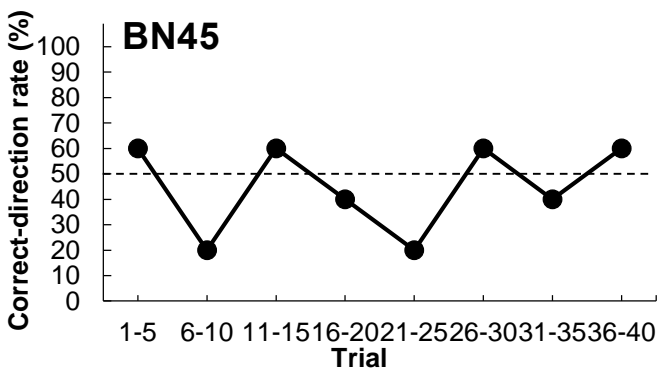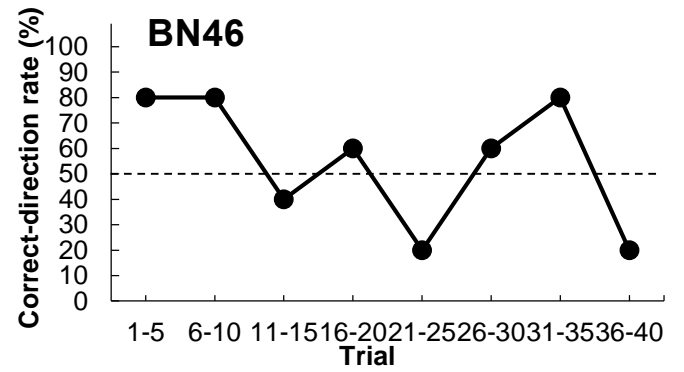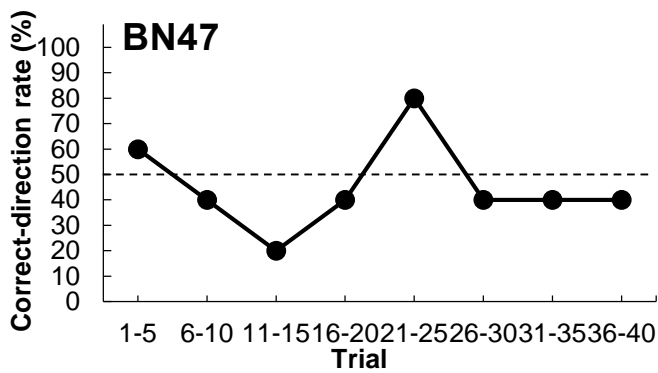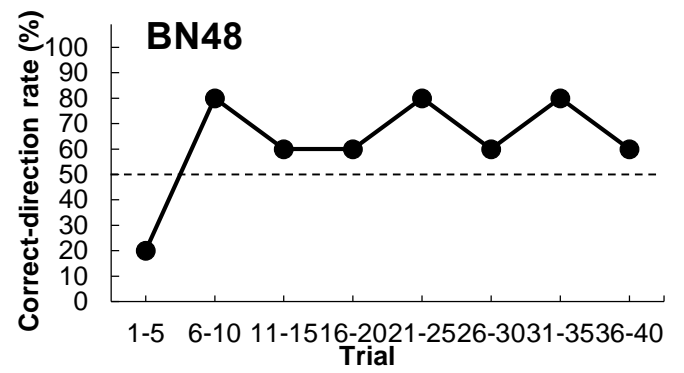

Supplement: S5 Fig — The broken line indicates chance level. (PDF) [file pone.0226569.s007.pdf]

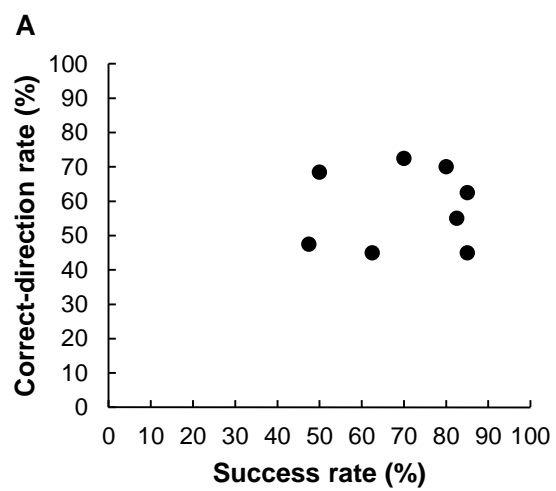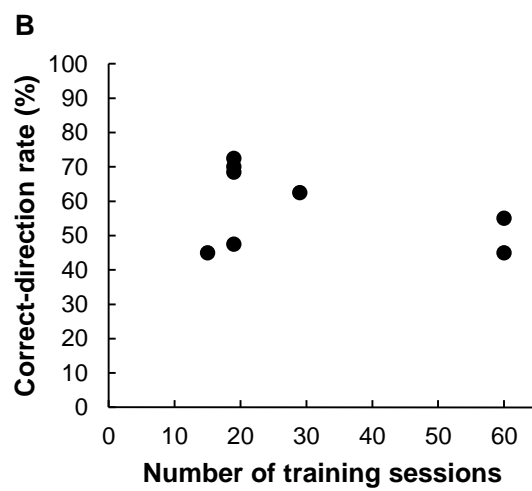

Supplement: S6 Fig — (A) Individual results on the relationship between the correct-direction rate and the success rate. The vertical axis shows the correct-direction rate in the positional discrimination test, and the horizontal axis shows the success rate on the last day of the rake-choice training. (B) Individual results on the relationship between the correct-direction rate and the number of training sessions. The vertical axis shows the correct-direction rate in the positional discrimination test, and the horizontal axis shows the number of sessions in the rake-choice training. (PDF) [file pone.0226569.s008.pdf]

**A**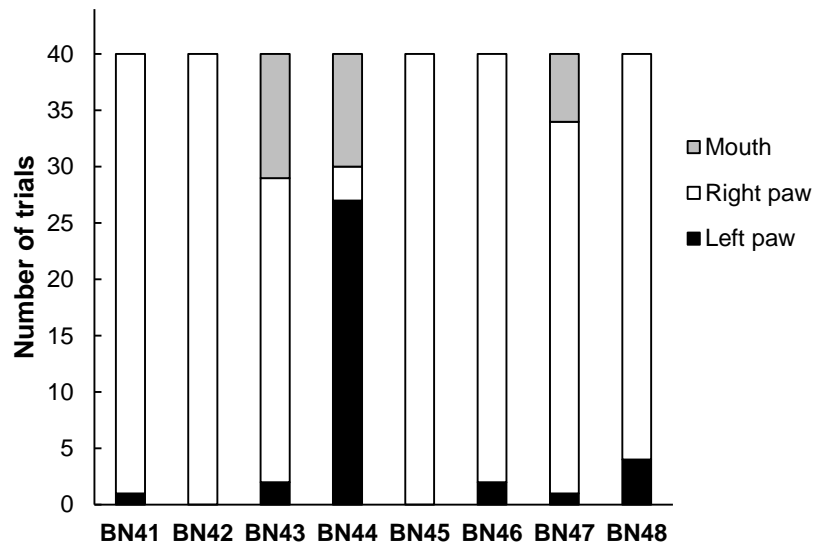**B**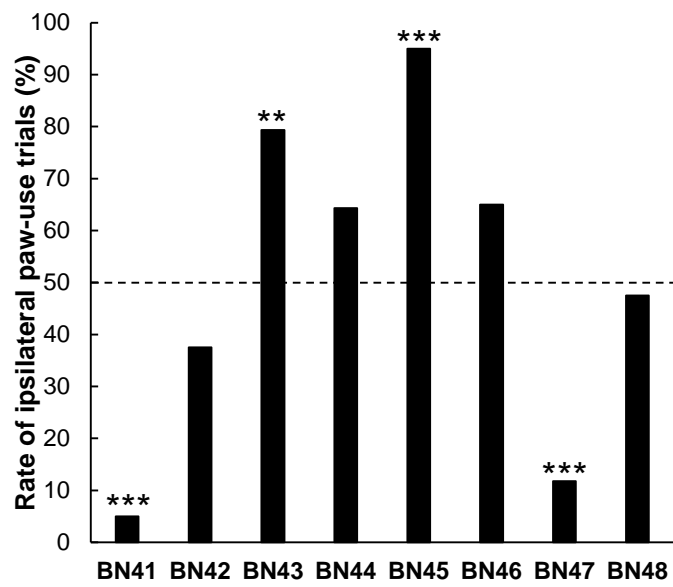**C**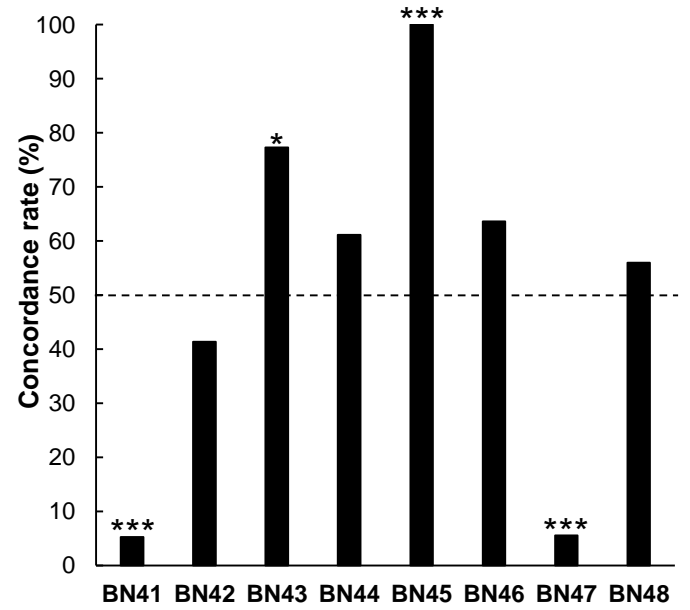

Supplement: S7 Fig — (A) Individual results for the number of trials in which each body part was used for the longest duration in the test. (B) The individual rates of ipsilateral paw-use trials in the test. Trials in which each rat used its paw for the longest duration were excluded from the analysis. The broken line indicates chance level (**p < 0.01, ***p < 0.001). (C) The individual concordance rates between the correct-direction trials and the trials in which the reward was on the same side as their paw used for pulling the rake for the longest duration in the test. Trials in which each rat used its paw for the longest duration were excluded from the analysis. The broken line indicates chance level (*p < 0.05, ***p < 0.001). (PDF) [file pone.0226569.s009.pdf]
